# Hollow Cholesteric Liquid Crystal Elastomer Fiber with Synergistically Enhanced Resilience and Mechanochromic Sensitivity

**DOI:** 10.1002/advs.202504487

**Published:** 2025-06-25

**Authors:** Wenwen Wang, Weijie Cheng, Feixia Liu, Kangyu Jia, Huihui Zhan, Chenguang Yang, Ke Liu, Qiongzhen Liu, Dong Wang

**Affiliations:** ^1^ Key Laboratory of Textile Fiber and Products (Ministry of Education) Hubei International Scientific and Technological Cooperation Base of Intelligent Textile Materials & Application Wuhan Textile University Wuhan 430200 China

**Keywords:** cholesteric liquid crystal elastomer, hollow fiber, mechanochromic, resilience, sensitivity

## Abstract

Mechanochromic cholesteric liquid crystal elastomer (CLCE) are unique anisotropic rubbers with programmable optical properties in response to external stimuli, bringing considerable interest in display, sensing, and smart textiles. However, creating mechanochromic CLCE fibers with high sensitivity, excellent resilience, and stable color‐changing reversibility is still a challenge. This work describes mechanochromic CLCE symmetric hollow fibers with excellent radial helix alignment prepared by solvent evaporation‐induced assembly and template assistance. The cylindrical symmetry renders the response identical in all directions perpendicular to the fiber axis. The hollow fibers show mechanochromic ability in the full visible spectrum, a large wavelength blueshift of 2.25 nm·%^−1^ under low strain. Excellent mechanochromic reversibility of 100 cycles and resilience are both devoted by cross‐linking structure in the wall layer, which also contributes to reversible orientation‐recovery of liquid crystal units during stretching‐recovery cycles. Numerical models and experimental measurements demonstrate the hollow structure enabled fibers to produce air pressure inside when being stretched, and the fiber wall layer is subjected to greater stress compared to solid fibers, thus achieving high mechanochromic sensitivity. In addition, hollow fiber can still change color when subjected to the expansion stress inside the cavity, and programmable information display in fabric prolongs its application in intelligent textiles.

## Graphical abstract

It describes hollow cholesteric liquid crystal elastomer fiber with radial helix prepared by solvent evaporation‐induced assembly and template assistance. The fiber show mechanochromic wavelength blueshift of 2.25 nm·%^−^^1^ under low strain. Excellent mechanochromic reversibility and resilience are devoted by cross‐linking in the wall layer. The hollow structure enabled fibers to produce air pressure inside with stretching, achieving high mechanochromic sensitivity.

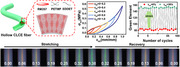

## 1 Introduction

Recently, mechanochromic materials have attracted considerable attention owing to their applicability in strain sensing, structural health monitoring, anticounterfeiting, and encryption.^[^ ^1^, ^2^, ^3^, ^4^ ^]^ According to the origination of colors, it can be broadly categorized into two types: pigmented color and structural color. Pigmented color is achieved through the selective absorption of incident white light by substances, and structural color arises from the interaction between periodic nanostructures and incident light.^[^ ^5^, ^6^, ^7^, ^8^, ^9^ ^]^ In contrast, structure color remains vivid under daylight and is free of photobleaching problems.^[^ ^10^, ^11^ ^]^ This mechanochromism is a direct consequence of the deformation‐induced changes in the periodic lattice constants of structurally colored materials.^[^ ^12^ ^]^ Cholesteric liquid crystal (CLC), which are chiral nematic liquid crystal (LC), are prominent as the structural colors, which can be easily programmed.^[^ ^13^ ^]^ The colors arise from the helical twisting of anisotropic rod‐shaped LC molecules along an axis perpendicular to the molecular director which in turn can be tuned by the concentration of the chiral dopant.^[^ ^14^, ^15^ ^]^ Cholesteric liquid crystal elastomer (CLCE), due to their mechanochromism, flexibility, and versatility, have become a hot topic in multi fields from flexible artificial skins to visualized sensors and smart biomimetic devices.^[^ ^16^, ^17^, ^18^, ^19^ ^]^ CLCE combines the elastic behavior of rubbers with the programmable optical properties of CLC. The distance over which the nematic director at each plane rotates completely (360°) is defined as the pitch (P). CLCE can exhibit selective reflection at a specific wavelength (𝜆) that varies with the pitch, in accordance with Bragg's law:^[^ ^20^ ^]^

(1) $$\lambda =n_{avg}\mathrm{Pcos}\theta$$

where 𝜃 is the angle of incident light and n_avg_ is the average refractive index of CLCE. For 𝜃 = 0, that is, in retroreflection configuration, the reflected wavelength has its maximum value 𝜆_0_ = *n_avg_* P. By adjusting the pitch, it is possible to control the reflection wavelength of CLCE over the whole visible spectrum. In addition, P will be altered if CLCE responds to mechanical‐stress‐inducing events, such as stretching, compression, and bending.^[^ ^21^ ^]^ And the reflection wavelength will change simultaneously. Upon deformation, the magnitude of the spectral shift is expressed as Δλ/λ = ΔP/P.

CLCE hasbeen widely investigated in 3D printing and thin film materials, etc., but CLCE fibers are rarely reported.^[^ ^22^, ^23^, ^24^ ^]^ However, mechanochromic fibers, due to their flexibility and weavability, are potential in mechanochromic smart textiles for healthcare, information encipherment, and sports, etc.^[^ ^25^, ^26^ ^]^ Geng and coworkers prepared ribbon‐like CLCE fibers with full visible spectrum mechanochromic response by dry spinning, but because of their non‐circular section, the surface color is differently observed from different radial angles.^[^ ^27^ ^]^ Although this problem was solved later to obtain the fibers with radial liquid crystal unit distribution and circular cross‐section, the resilience and mechanochromic sensitivity still need to be further investigated and improved.^[^ ^28^ ^]^ Excellent mechanical stability, resilience, and high sensitivity are of paramount significance for the application of advanced smart textiles.

Therefore, high sensitivity through color change means that weak force or low strain can make the pitch of CLCE change greatly to achieve a large spectral shift, and therefore obvious color change can be seen by the naked eye.^[^ ^29^, ^30^ ^]^ As reported, the capability of CLCE with a large Poisson's ratio (ν>0.5) is one way to achieve high sensitivity.^[^ ^31^, ^32^ ^]^ The essence of large Poisson's ratio is that when CLCE is subjected to uniaxial stretching, the pitch perpendicular to the strain direction is greatly compressed, thus acquiring a broad reflective band shift and realizing a significant change in color. However, considering the utility of the material, the large Poisson ratio should be regulated in concert with the material modulus, resilience, and the self‐assembly process of the liquid crystal units. Additionally, the liquid crystal units in CLCE are rigid rod structures, which will inevitably orientated along the external force. To make CLCE fibers have excellent reversible response, that is, both macroscopical physical deformation of the fiber and liquid crystal units should be synchronized, and can be reversible under external forces with multiple cycles. Therefore, producing high‐quality mechanochromic CLCE fibers remains challenging.

Herein, we proposed a new and facile solvent evaporation‐assisted template method to prepare CLCE hollow fibers. The oligomer solution was first prepared with liquid crystal monomer, chain extender, cross‐linker, and chiral dopant. The concentration of chiral dopant was regulated to produce fibers with original colors of red, green, and blue. With the solvent evaporation, liquid crystal units self‐assembled on the circular surface of the template, thus obtaining cylindrically symmetric hollow fibers. By regulating crosslinker concentration, the mechanochromic hollow fiber with excellent mechanical properties could achieve its color change in the full visible spectrum and have a large wavelength shift under low strain, as well as excellent mechanochromic reversibility. Through theoretical simulations and experimental measurements, we also investigated the effect of the hollow structure on mechanochromic sensitivity. In addition, we investigate the application of the hollow fiber in the intelligent fabric for programmable information display, which was achieved by expansion stress inside the cavity.

## 2 Results and Discussion

### 2.1 Fabrication and Characterization of Hollow CLCE Mechanochromic Fibers

**Figure 1:**
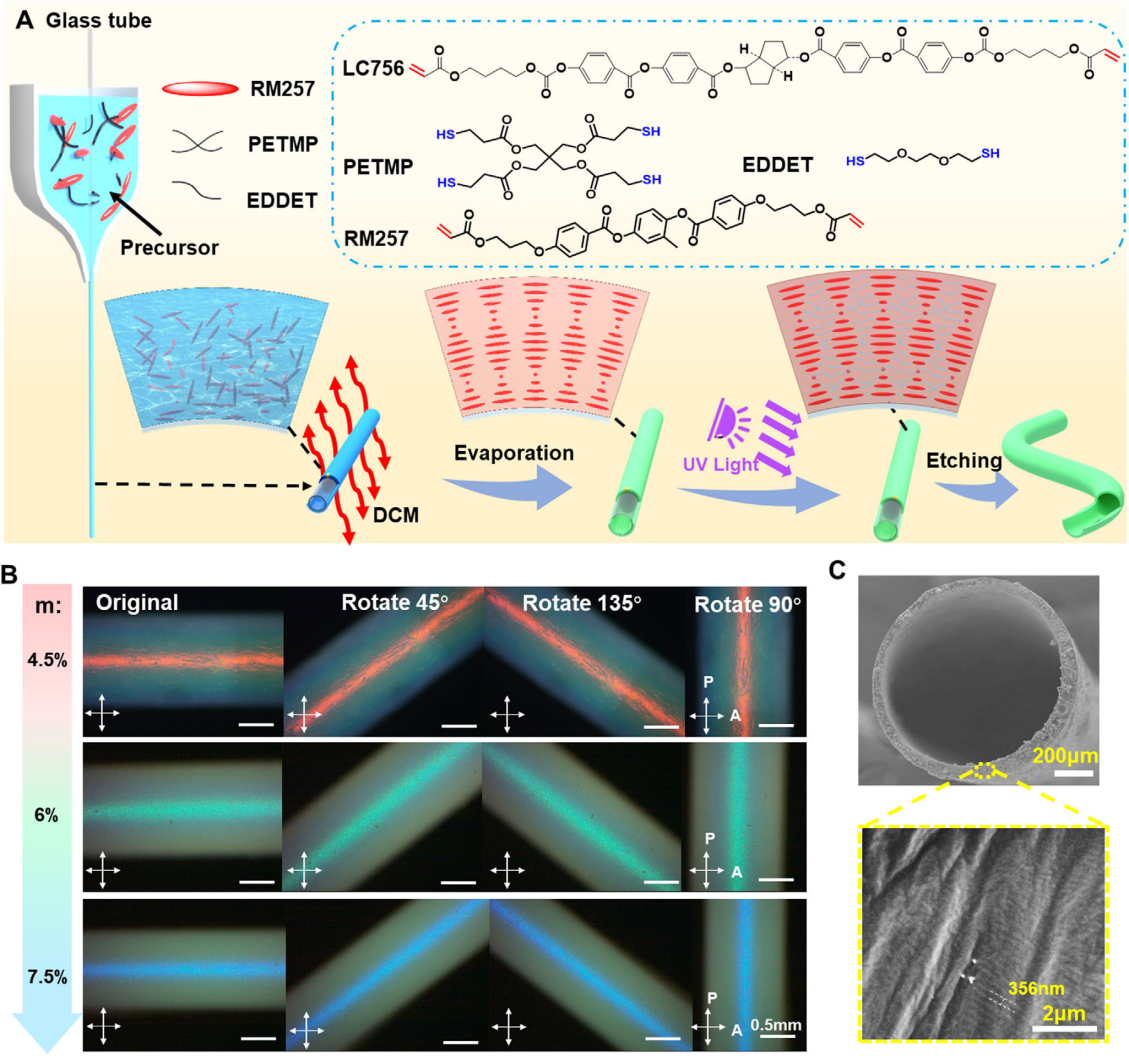
Fabrication and characterization of hollow CLCE mechanochromic fiber. A) Schematic fabrication procedure of hollow CLCE mechanochromic fiber: RM257 as mesogen, EDDET as chain extender, and PETMP as crosslinker. B) The reflected POM images of hollow CLCE mechanochromic fiber with different original colors of red, green, and blue fabricated by different m values. C) SEM images of hollow CLCE mechanochromic fiber with original red color.

**Figure** **1**A schematically illustrates the fabrication process of hollow CLCE mechanochromic fiber, where a CLCE mechanochromic fiber with a hollow structure and self‐assembled helical nanoarchitecture was first prepared using a cylindrical capillary glass tube model and evaporation‐induced self‐assembly method. The compositions of monomers, chain extender, and crosslinker were optimized to afford the hollow CLCE fibers to show different original color spectrums upon UV light cross‐linking. In the fabrication process, the precursor with low crosslinking density and optimal viscosity was prepared by Michael addition reaction, in which the CLC monomers could quickly self‐organize into helical nanostructure by solvent evaporation. The resulting photonic patterns exhibit vivid structural colors, which are further irradiated by UV light to trigger the photoinitiated crosslinking. It is found that the solution viscosity of the precursor is an important parameter for fiber forming due to Plateau‐Rayleigh instability.^[^ ^27^, ^28^, ^29^, ^30^, ^31^, ^32^, ^33^, ^34^ ^]^ Low viscosity renders it too fluid, causing a noncontinuous surface or longer self‐assembly time. Solvent‐evaporation induced driving force is insufficient for the precursor to self‐assemble into a uniform helical arrangement if high viscosity. And the thickness of the fiber wall can be controlled according to the precursor viscosity and coating speed. In this work, to control the mechanical properties and mechanochromic sensitivity synergistically, the wall thickness of fibers with different original colors was ≈100 µm.

By changing the concentration of the chiral dopant, we easily tune the original reflection wavelength (𝜆_0_) of the fiber from IR to blue as desired. Figure 1B displays the vivid POM reflection red, green, and blue colors under crossed polarizers when the fibers were rotated to the direction of 45°, 90°, and 135° relative to both polarizers. The fibers are uniform and circular in diameter and show intense selective reflection at the top. Between crossed polarizers with a λ plate inserted, the red, green, and blue colors remain, almost unaffected by fiber rotation, confirming the Bragg reflection from well‐aligned CLCE hollow fibers with circularly polarized properties. The helical nanostructures were further confirmed by SEM images of the cross‐section micromorphology of the fiber as shown in Figure 1C, where the helical pitch of the red hollow fiber was found to be ≈356 nm. It produces light reflection with the maximum wavelength of ≈570 nm based on Equation (2):^[^ ^35^ ^]^

(2) $$\lambda_{\max}=n_{\mathrm{avg}}P$$

where P is the helical pitch, and n_avg_ ≈ 1.6 is the measured average refractive index of red hollow fiber, which is like the reported result.^[^ ^27^, ^36^ ^]^ There is a difference between the wavelength calculated by Equation (2) and the surface color of red hollow fiber, which is attributed to the measurement error of n_avg_.

**Figure 2:**
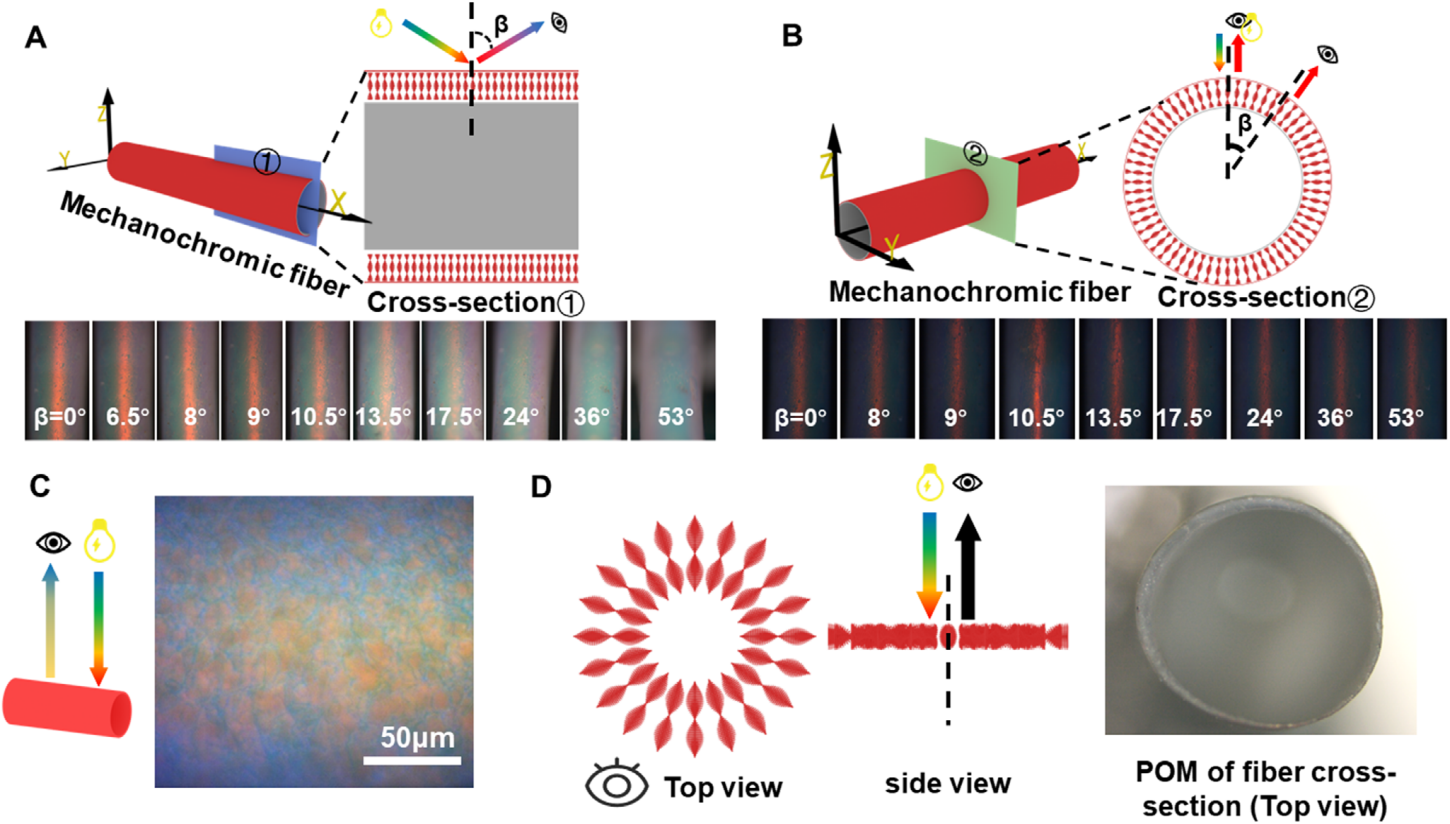
Characterizations of assembled helical morphology in hollow CLCE fibers. A,B) Drawings schematically show the experiment configuration for different cross‐sections, and reflected POM images of the fibers observed by varying angles β. C,D) Schematic measurements and reflection POM image observed perpendicular to the fiber axis and cross‐section.

To further investigate the assembled morphology of hollow fibers, characterizations of assembled helical morphology in hollow CLCE fibers can be seen in **Figure** **2**. As reported in other work,^[^ ^28^ ^]^ we place the fiber with red λ_0_ on a glass slide for investigation by POM as a function of the angle β from the imaging plane. It can be seen from Figure 2A that the red color is observed perpendicular to the fiber axis (β = 0) but the reflection intensity decreases gradually by increasing β. However, the radial color remains the same regardless of β in Figure 2B, confirming the excellent radial orientation of the helical axis throughout the fiber. In addition, the radial orientation of the helical axis can also be demonstrated by that the color can be seen at the surface of the fiber in Figure 2C and it doesn't display color from the cross‐section in Figure 2D even though both are observed by POM. The appearance gives additional proof of radial helix throughout the fiber.

### 2.2 Mechanochromic Response Sensitivity, Reversibility and Fiber Resilience

**Figure 3:**
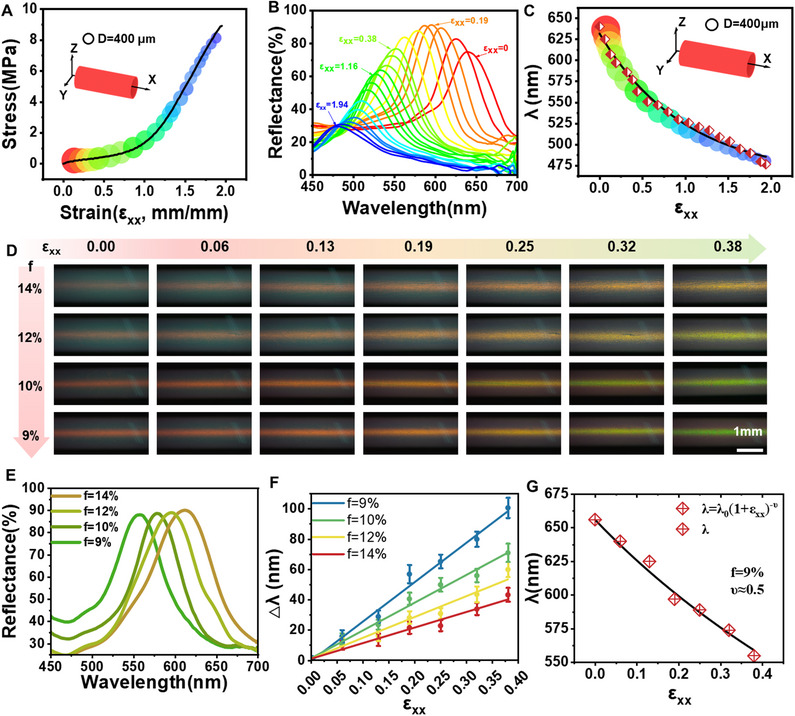
A) Stress–strain curve of red retroreflection hollow CLCE mechanochromic fiber under uniaxial stretching when f = 9%. Symbol size represents fiber width (scale indicated by a ring of diameter D). B) Spectra of the selectively reflected light corresponding to the strain of 0.00 to 1.94. C) Central retroreflection wavelength *λ* versus engineering strain ε_xx_. D) Photographs of initially red‐reflecting hollow fibers with different f values under uniaxial mechanical stretching (from 0.0 to 0.38). E) Reflection spectra of the hollow fiber fabricated by f = 14%, 12%, 10%, and 9% at ε_xx_ = 0.38, respectively. F) Reflective wavelength shifts Δλ as a function of uniaxial tensile strain ε_xx_. G) Retroreflection wavelength 𝜆 versus ε_xx_. The black line is the best fit for Equation (3).

In this work, the effect of chemical structures on the mechanical properties of hollow CLCE mechanochromic fibers was investigated by regulating the molar ratio of crosslinker (PETMP) to chain extender (EDDET). The stress–strain curves can be seen in **Figures** **3**A and S1 (Supporting Information). Regulation of the chemical composition facilitates the acquisition of fibers with different modulus, strength, and elongation at break. The strength and modulus of the fiber were gradually decreased with the decrease of crosslinker (PETMP). When the crosslinker ratio is 18% or 8%, the fibers are too rigid or soft for practical utility. Accordingly, the fibers with the crosslinker of 9%, 10%, 12%, and 14% were studied further. First, the mechanochromic response was investigated by uniaxial stretching. As shown in Figure 3A, the elongational stress σ_xx_ of red retroreflection hollow CLCE mechanochromic fiber is plotted as a function of engineering strain ε_xx_, using circular symbols with the color of reflection at each ε_xx_ value and a radius shrinking like the fiber width. When f = 9%, the fiber possesses excellent mechanical properties and the stress at break can reach up to ≈9 MPa. The mechanochromic behavior and recovery can be seen in Video S1 (Supporting Information).

To quantify the mechanochromic response, the reflection spectra were also measured. The full visible spectrum is available for revealing the magnitude of the elongational strain to which the fiber is subjected. The reflection peak wavelength continuously shifts from 639 to 477 nm as a function of strain (Figure 3B). It should be noted that the highest peak intensity first rises before falling. It might result from the unwinding of the helix and the decreasing pitch of cholesteric nanostructures, which was also demonstrated in the reported work.^[^ ^22^ ^]^ Additionally, central retroreflection wavelength λ versus engineering strain ε_xx_ (ε_xx_ = Δ*l_x_/l_x0_*) were characterized, where Cartesian coordinate axis x is the fiber's long axis, *l_x0_* is the original length and Δ*l_x_* is the extension along the fiber.^[^ ^27^ ^]^ At each strain level, reflection wavelength *λ* can be extracted from the reflection peak in the spectra and it is plotted as a function of strain, which is shown in Figure 3C. Although the reflection wavelength range after the fiber is stretched is like that reported in the literature, the color of the fiber can be changed to green at a smaller strain of 0.38, which is much lower than that reported before.^[^ ^6^, ^22^, ^23^, ^30^ ^]^ In other words, under the same uniaxial strain, the fiber has a larger reflection wavelength shift of 2.25 nm·%^−1^ (wavelength shift per strain), which is more favorable to visual observation.

In addition, the effect of crosslinking on the mechanochromic sensitivity of CLCE hollow fibers was also investigated by varying the molar ratio of PETMP to EDDET from 9% to 14%. The swelling experiments using DCM as a solvent were carried out to detect the gel content. It shows in Figure S2 (Supporting Information) that the gel content increases gradually with the crosslinker ratio, which indicates the crosslinking density of hollow fiber increases from 9% to 14%. In Figure 3D, when the crosslinking density decreases, the surface color of the fiber turns green faster with the stretching, which means the mechanochromic sensitivity of the hollow fiber increases. DSC curves indicate that these CLCE hollow fibers possess LC‐to‐isotropic transition temperature (T_i_) at 45–68 °C (Figure S3, Supporting Information), respectively. With the decrease of crosslinker, the color change of the hollow fiber at the same uniaxial strain is more obvious. Pitch changes lead to color changes during stretching. The increase of crosslink brings a high modulus of the wall of hollow fibers, thus resulting in low sensitivity. It can also be seen from Figure 3E and Figure S4 (Supporting Information) that the reflection spectra of hollow fibers at ε_xx_ = 0.38 display an obvious blue shift with the decrease of crosslinker. We plot the wavelength shift (Δλ) versus uniaxial stretching strain (Δλ ∼ ε_xx_), as shown in Figure 3F. The slopes of the fitted curves also indicate that the fibers become insensitive with the increase of crosslinker. When f = 9%, the fiber possesses the optimal mechanochromic sensitivity.

When the hollow fibers are stretched along the fiber axis, the fiber wall is compressed and the CLCE pitch decreases.^[^ ^37^ ^]^ We analyze the mechanochromic response considering a Poisson's ratio (ν) that relates the strain ε_zz_ along the radial direction (thus along the helix axis) to the strain ε_xx_ along the fiber axis. According to the reported work,^[^ ^28^ ^]^ we can thus fit a function

(3) $$\lambda =\lambda_{0}{\left(1+\varepsilon_{xx}\right)}^{-\nu }$$

to obtain the Poisson's ratio (ν) from the mechanochromic data, assuming constant ν. By fitting Equation (3) to the mechanochromic reflection spectra, when the content of crosslinker (f) is 9%, ν of the fiber is closer to 0.5, which is ν of isotropic rubbers (Figure 3G; Figure S5, Supporting Information). However, when the crosslinker increases from 9% to 12%, ν decreases gradually from 0.5 to 0.31, which is higher than the ν_xz_ = 2/7 obtained by Cicuta et al. for pitch compression along $\hat{x}$ of flat CLCE elongated along $\hat{z}.$ ^[^ ^37^ ^]^ When the crosslinker content is 14%, the ν decreases to the lowest 0.24, which is ascribed to the elasticity decreasing of the hollow fiber. As reported before, Yang et al. prepared the inflating main‐chain chiral nematic liquid crystalline elastomer (MCLCE)membrane including a thick supporting layer and a much thinner mechanochromic layer, and the large disparity in thickness of the thin MCLCE membrane versus the supporting layer affords the instantaneous and robust response of structural coloration to the applied pressure so that the in‐plane strain is applied to the MCLCE membrane under out‐of‐plane inflation.^[^ ^31^ ^]^ Herein, although the largest ν is close to that of solid CLCE fiber,^[^ ^28^ ^]^ the liquid crystal elastomer layer is only a thin shell (≈100 µm), the applied strain acts on the shell, and the shell is compressed to a greater degree. It is coincident with that of cephalopods by decreasing the inter‐lamellae space to dynamically tune the color and brightness of their skin for camouflage and communication.^[^ ^38^ ^]^ Moreover, the effect of the hollow fiber wall thickness on the mechanical and mechanochromic properties was also investigated, and the result can be seen in Figure S6 (Supporting Information). It displays that the strength and elongation at break decrease if the wall is thinner and the mechanochromic sensitivity doesn't decrease. The reflectivity of the fiber with a thinner wall is lower, which is attributed to the light easily passing through the thinner fiber.

**Figure 4:**
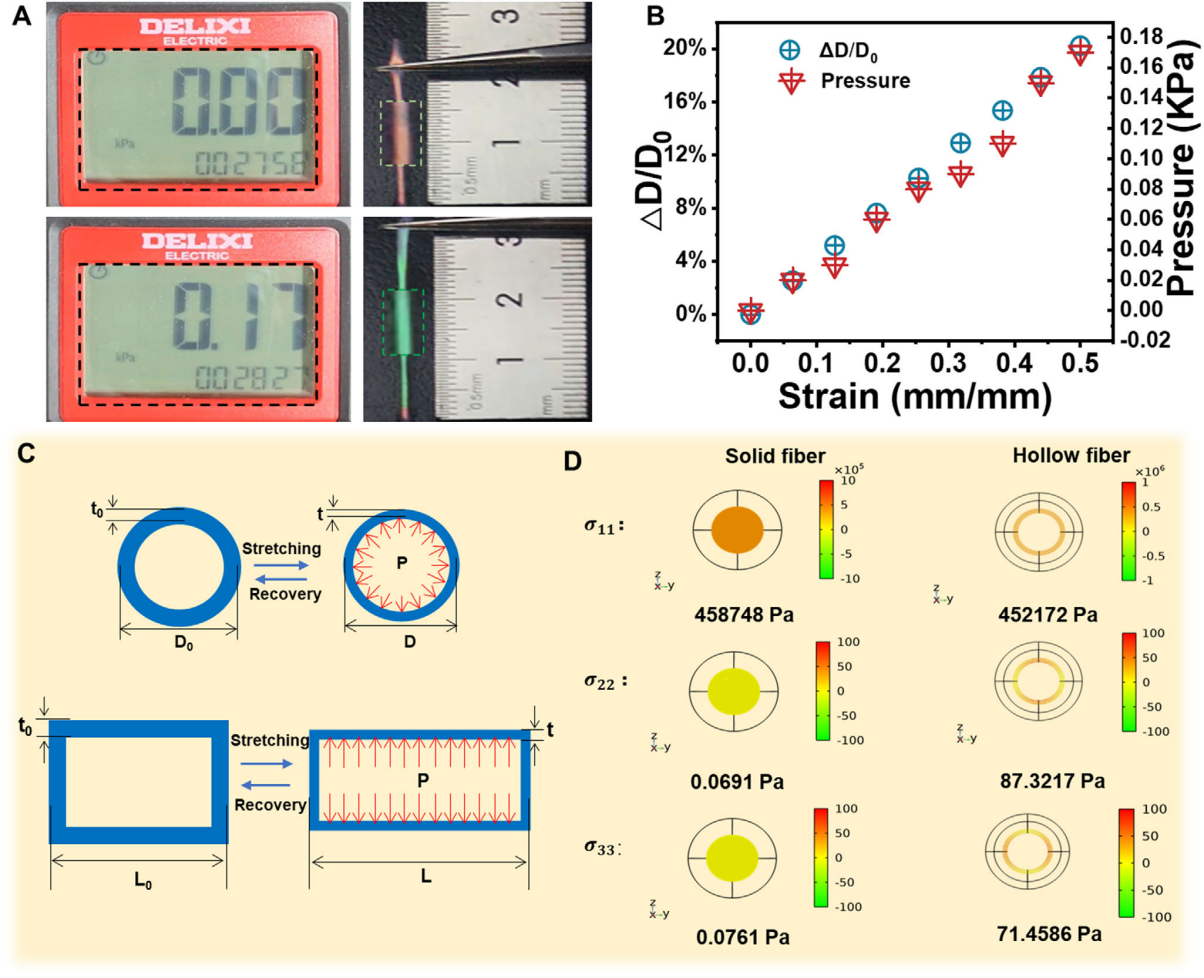
A) The pressure in the CLCE hollow mechanochromic fiber before and after stretching. B) The diameter and inner pressure of CLCE hollow mechanochromic fiber versus uniaxial strain. C) The schematic illustration of the CLCE hollow mechanochromic fiber change before and after stretching (t_0_, t: the wall thickness of the original and stretched fibers; D_0_, D: the external diameter of the original and stretched fibers; L_0_, L: the length of the original and stretched fibers. P: inner pressure of the hollow fiber.). Top: the radial cross‐section of fiber; Down: the axial cross‐section of fiber. D) Calculation results of numerical models by Finite Element Method (FEM) for both solid and hollow fibers. σ_11_: Axial stress component; σ_22_: Radial stress component; σ_33_: Circumferential stress component.

Additionally, we investigated the variation of inner pressure and external diameter of the CLCE hollow mechanochromic fiber under the uniaxial strain. In **Figure** **4**A, one end of the fiber was connected to the barometer hermetically, and the other end was clamped by the tweezers during stretching. The inner pressure of the hollow fiber is 0.17 kPa when the uniaxial strain is 0.5. In Figure 4B, we set the change of external diameter as ΔD = D_0_‐D. As the strain increases, the external diameter decreases gradually, and the inner pressure increases simultaneously. As illustrated in Figure 4C, we believe that, as stretching occurs, the wall thickness of fiber decreases. Meanwhile, the air pressure inside the fiber applies pressure to the wall. These effects cause the pitch within the fiber wall to be greatly compressed, resulting in higher sensitivity. Moreover, numerical models have been established by Finite Element Method (FEM) for both hollow fiber in this work and solid fiber in the reported work.^[^ ^28^ ^]^ The geometric models of solid and hollow fibers can be seen in Figure S7 (Supporting Information). Results in Figure 4D and Figure S8 (Supporting Information) show that, under external loading, the stress tensor components in the local coordinate system of the mid‐sectional plane for the solid fiber and hollow fiber are different. When the axial strain is 50%, the axial stress component (σ_11_) of the solid fiber is a little larger than that of the hollow fiber of this work. However, the radial stress component (σ_22_, 87.3217 Pa) and circumferential stress component (σ_33_, 71.4586 Pa) of the hollow fiber are 1263 and 939 times those of the solid, respectively. It confirms that under the same strain, the hollow fiber is subjected to greater stress in the radial direction, causing the liquid crystal helix pitch to be compressed to a greater extent, resulting in a larger spectral shift and more sensitive color change.

**Figure 5:**
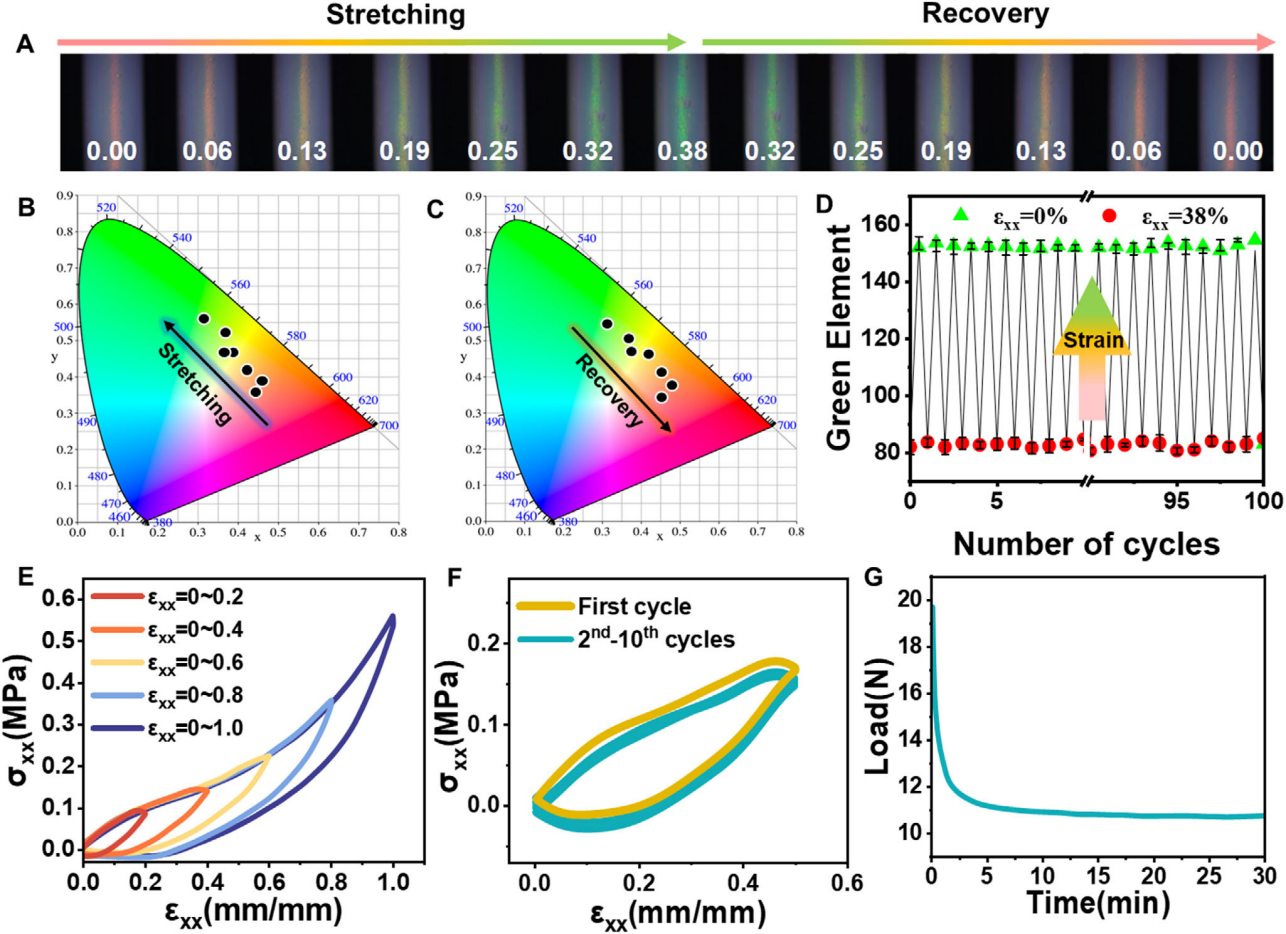
A) Uniaxial stretching and recovery behavior of hollow CLCE mechanochromic fiber under length strain from 0.00 to 0.38. B,C) Change in the reflective color under a length strain of 0.00 to 0.38 on the CIE 1931 color space during stretching and recovery. D) Changes of a green element in the CIE xy chromaticity diagram during 100 times stretching‐recovery cycles under a length strain of 0.00 to 0.38. E) Hysteresis curves of hollow CLCE mechanochromic fibers with f = 9% under different strains of 0.2, 0.4, 0.6, 0.8, and 1.0. F) Hysteresis curves of hollow CLCE mechanochromic fibers with f = 1.0 were obtained from ten times load and unload tests at room temperature up to strain of 0.5. G) The stress relaxation performance of the red retroreflection fiber with f = 9%.

The mechanochromic reversibility is investigated by fiber stretching and recovery. As shown in **Figure** **5**A, when the fiber is relaxed, the color can recover again. As shown in Videos S2 and S3 (Supporting Information), the response is immediate, fully reversible, and visible to the naked eye. It can be seen from Figure 5B,C that the fibers show the same color in CIE 1931 at the same strain, either during stretching or recovery. It indicates that the pitch in fiber can recover immediately if the strain recovers according to Bragg's law.^[^ ^39^ ^]^ To evaluate the long‐term durability under realistic usage conditions, hollow CLCE mechanochromic fibers with f = 9% were measured by cyclic stretching for strains up to ε_xx_ = 0.38 and then subjected to 100 cycles of ε_xx_ = 0→0.38→0. As shown in Figure 5D, there is no obvious change in the green element of the color in CIE 1931 at a strain of 0.38 beyond experimental variability, and the color recovers at a stain of 0.0. The mechanochromic response is practically intact. These data clearly show that it is realistic to use the fibers in smart textiles where they will be stretched and relaxed under low strain repeatedly.

After repeated stretching of the fiber, the color can maintain stable change, which is also related to the mechanical properties of the fiber, especially its resilience. We perform cyclic tensile tests to evaluate the resilience of the fiber. First, cyclic tensile tests of the fiber are performed with gradually larger strains from 0.2 to 1.0. In these sequential cyclic tensile tests (Figure 5E), there is no waiting time between two consecutive loadings. Clearly, the fiber possesses good resilience owing to the small hysteresis loop.^[^ ^7^, ^40^, ^41^ ^]^ Subsequently, the resilience of the fiber is also assessed via cyclic tensile tests. When the sample is successively stretched up to the preset strain of 0.38 and released, stress–strain curves are detected between the following and previous cycles. Figure 5F shows that the stress–strain curves almost coincide each time and the hysteresis loss in Figure S9 (Supporting Information) hasn't gradually increased with the increasing stretches. It demonstrates that high resilience and reversible color stability can be achieved through the formulation design of CLCE with chemical crosslink and the cross‐section design of the fibers. The stress relaxation of the red retroreflection fiber in Figure 5G shows that it can relax ≈45% of its stress within 5 min and then keep the stress stable, which displays the characteristic of cross‐linked elastic CLCE.^[^ ^42^ ^]^ In addition, we sewed up the fiber into the fabric (Figure S10, Supporting Information), the fabric displays strain sensing by the color changing of mechanochromic CLCE fibers under stretching, and the fiber also can recover immediately once the external force is released.

### 2.3 Morphological Evolution

**Figure 6:**
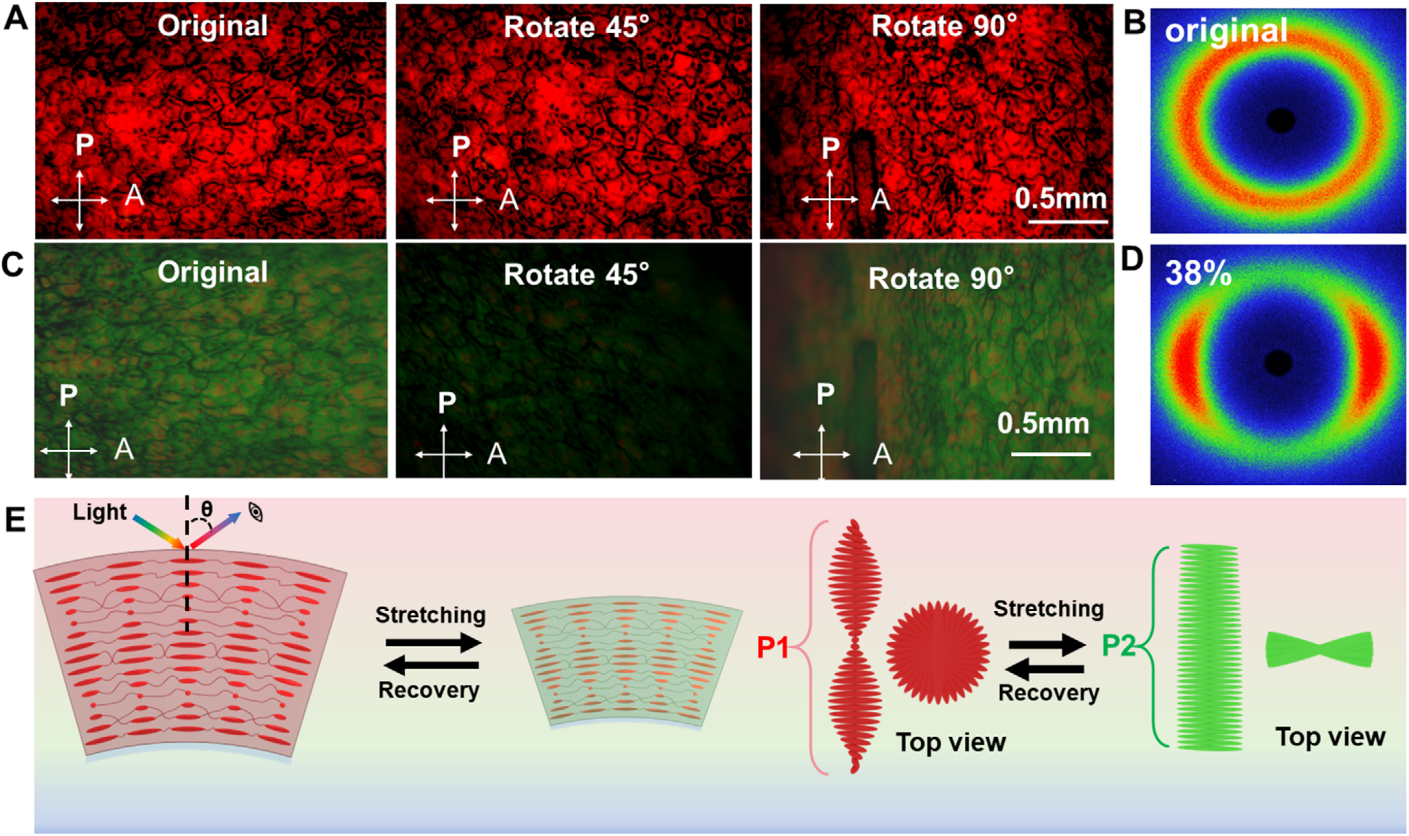
A,C) POM images of the original red‐reflecting CLCE hollow fiber and the 38% strained CLCE hollow fiber at different angles. B,D) The corresponding WAXS patterns of monodomain structure in the original red‐reflecting CLCE hollow fiber and strained polydomain structure subjected to stretching relative to the fiber axis. E) Schematic depictions of strain‐induced deformation and eventually unwinding of the cholesteric helix viewed parallel or perpendicular to the helix axis.

To further verify the microstructural changes of the CLCE hollow fibers upon uniaxial stretching, POM images are acquired while rotating the sample at 45° intervals under perpendicular stretching at a strain of 0.38 (**Figure** **6**A,C; Figure S11, Supporting Information). The unstrained CLCE hollow fiber exhibits nearly uniform birefringence regardless of the observation angle. However, the birefringence of the strained CLCE hollow fiber gradually disappeared‐especially at 45°‐with increasing strain. These results suggest that all the mesogens in the CLCE hollow fiber tend to align in the direction of the applied uniaxial strain and lose the helical structure.^[^ ^43^, ^44^ ^]^ To further investigate the microstructural changes of the CLCE hollow fibers during stretching, wide‐angle X‐ray scattering (WAXS) analysis is performed in the normal‐to‐plane geometry under original and stretched conditions, as summarized in Figure 6B,D. WAXS patterns change from a ring to a pair of arcs along the transverse direction, indicating the polydomain structure in the initial red‐reflecting CLCE hollow fiber and the monodomain structure in the stretched blue‐reflecting CLCE hollow fiber, which further confirms that the liquid crystal director predominantly aligns along the direction of mechanical stretching.^[^ ^42^, ^45^, When the CLCE hollow fiber was uniaxially stretched, its helical structure was untwisted and gradually transformed into a stratified structure. We schematically depict the changes of helix in the wall of hollow fiber, as shown in Figure 6E. Precisely because of the existence of moderate crosslinking, the fiber possesses excellent resilience, and the mesogen orientation is restored quickly with excellent color recovery.

### 2.4 Programmable Information Displays

**Figure 7:**
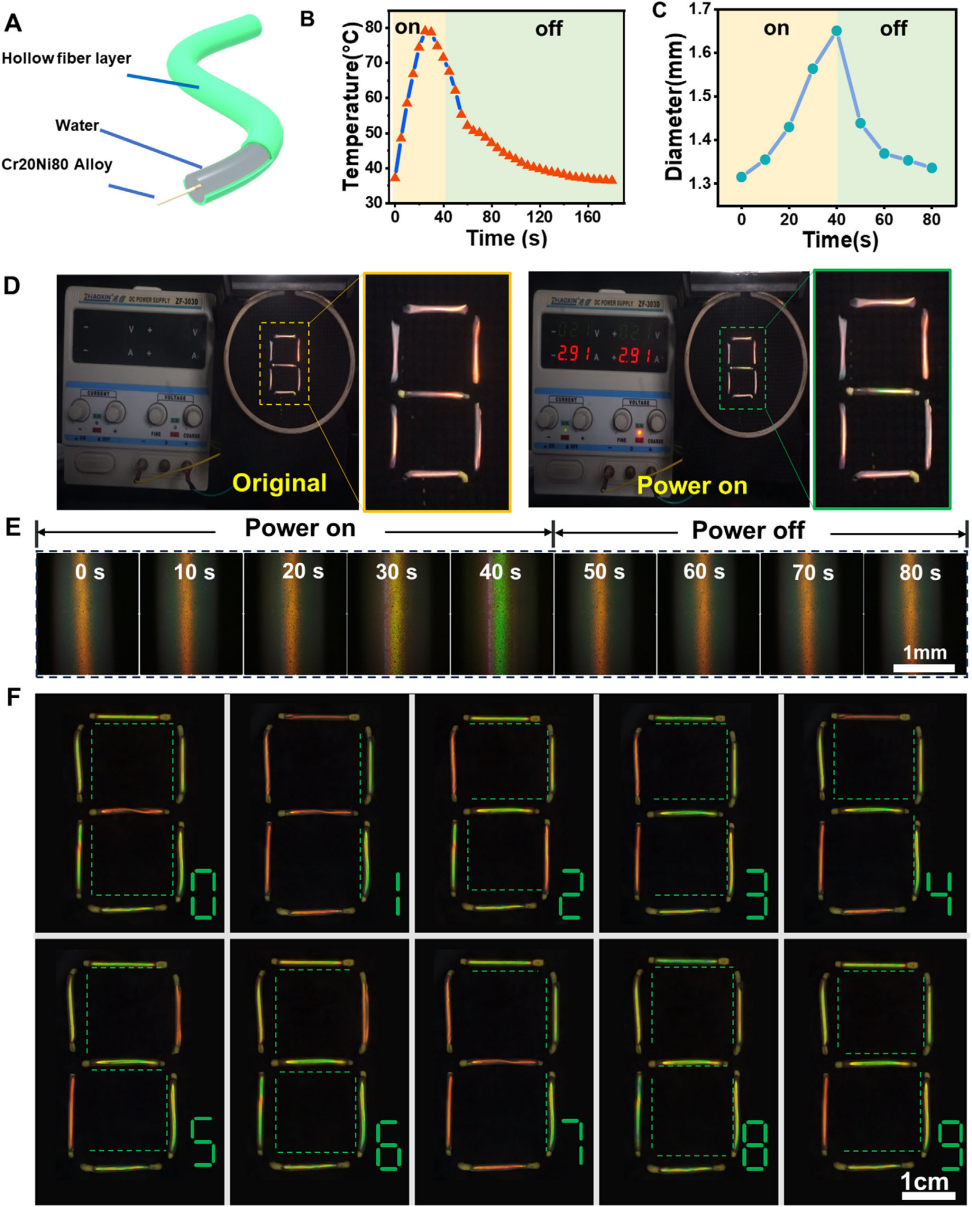
A) The schematic illustration of the composite fiber. B,C) The surface temperature and the external diameter of the composite fiber versus time for power on and off. D) The fiber images before and after being loaded voltage. E) The color evolution of fibers versus time for power on and off. F) The programmable information is displayed on the fabric.

Textile displays with flexibility and breathability like regular clothes could effectively fit human skin dynamically while being comfortable and serve as human‐computer interfaces, allowing users to interact with electronic devices.^[^ ^46^, ^47^ ^]^ In addition to the above mentioned that fibers can change color under stretching to detect and sense the stress or strain, the hollow fiber in this work also has the advantage that it can load the stress from the inside to make it expand, and the fiber wall is compressed to change color. Therefore, we explore its application in information display. As shown in **Figure** **7**A, first, the Cr20Ni80 alloy wire was inserted into the hollow fiber and then one end of the hollow fiber was sealed with UV curing adhesive. Then water was injected into the fiber with a syringe, and the remaining end of the hollow fiber was also sealed with UV curing adhesive. Due to the softness of the hollow fiber and Cr20Ni80 alloy wire, the composite fiber can be woven into the fabric and coded information. In Figure 7B,C and Figure S12 (Supporting Information), when the voltage is loaded at both ends of the composite fiber, due to the electrothermal effect of Cr20Ni80 alloy wire, the surface temperature of the fiber can reach up to 81.5 °C after 25s, and the water in the fiber is heated, so that the expansion of hollow mechanochromic fiber increases its diameter due to the expansion of its internal water (Figure S13 and Video S4, Supporting Information), the fiber wall is compressed, and the pitch is reduced, thus changing color.

As illustrated in Figure 7D,E, when the voltage is loaded for the 40s, the surface color of the fiber can turn green, and after the power is turned off, the color can be completely recovered, which also demonstrates that the hollow CLCE mechanochromic fiber has excellent color reversibility and sensitivity. Additionally, the reflection spectra of the fiber surface under varying temperatures are shown in Figure S14 (Supporting Information). The reflection wavelength can gradually recover to the original value when the power is off. It is worthily noted that to exclude the influence of the thermal expansion of the hollow fiber on its surface color, we only heated the fiber, and thus investigated its thermal expansion effect. As can be seen from Figure S15 (Supporting Information), the fibers cannot change color only by heating, which is different from the result reported in the literature.^[^ ^28^ ^]^ It may be attributed that the hollow CLCE mechanochromic fibers here have high modulus because of crosslinked structure and the temperature cannot change their pitch and change color. To exploit the potential application, we embed multiple, independent composite fibers in fabric for multiplexed coloration. A seven‐segment display consisting of seven fibers is fabricated in a small area, where different coloration units are linked to voltage to display one‐digit numbers from “0” to “9” (Figure 7F). Accordingly, it is because of the hollow mechanochromic fibers with high sensitivity, excellent resilience, and color reversibility prepared in this work that expands the application of mechanochromic materials in intelligent textiles. The pixelated color control gives us the confidence to attempt camouflage or information encrypting in future work.

## 3 Conclusion

In summary, we have demonstrated a straightforward strategy to prepare mechanochromic CLCE hollow fibers by solvent evaporation and template assistance. Mechanochromic fibers, with different original retroreflection colors and radial spiral director, have been obtained due to the regulation of chiral dopant and cylindrical template. The cylindrical symmetry renders the response identical in all directions perpendicular to the fiber axis. By regulating crosslinker concentration, the hollow fibers show strong mechanochromic ability in the full visible spectrum, a large wavelength blueshift of 2.25 nm·%^−1^ under low strain and excellent mechanochromic reversibility of 100 cycles, which is more advantaged than those of elastomer‐core liquid crystal fibers in the reported works.^[^ ^48^ ^]^ We explain this by the cross‐linking structure in the wall layer devoted to the excellent resilience of the fibers and reversible orientation‐recovery of the liquid crystal units in the stretching‐recovery cycles. However, compared with solid fiber, the mechanical strength of the hollow fiber is lower and the stability in complex environments may need to be improved. Numerical models and experimental measurements demonstrated that the hollow structure enabled the fibers to produce air pressure inside when being stretched, and the fiber wall layer is subjected to greater stress compared to the solid fibers, thus achieving high mechanochromic sensitivity. In addition, the hollow fiber can still change color when subjected to the expansion stress inside the cavity, and a programmable information display in the fabric was also carried out, which prolonged the application of mechanochromic CLCE in intelligent textiles.

## 4 Experimental Section

### Materials

1,4‐Bis‐[4‐(3‐acryloyloxypropyloxy) benzoyloxy]‐2‐methylbenzene (RM257, purity: 98.82%) and pentaerythritol tetra(3‐mercaptopropionate) (PETMP, purity: 95%) were purchased from Bidepharm. The chiral dopant (3R, 3aS, 6aS)‐hexahydrofuro[3,2‐b] furan‐3,6‐diyl bis(4‐(4‐((4‐(acryloyloxy) butoxy) carbonyloxy) benzoyloxy) benzoate) (LC756, purity: 90%) was purchased from Adamas‐beta. 2,2′‐(Ethylenedioxy) diethanethiol (EDDET, purity: 97%), 2,2‐dimethoxy‐2‐phenylacetophenone (I‐651, purity: 99%), and dipropylamine (DPA, purity: 99%) were purchased from Aladdin. Dichloromethane (DCM, AR) was purchased from Sinopharm.

### Precursor Synthesis and Fabrication of Hollow CLCE Mechanochromic Fibers—Precursor Synthesis

The precursors were prepared based on Michael addition reaction according to the publications reported previously.^[^ ^15^, ^33^ ^]^ The following outlines a standard procedure for the synthesis of precursors: RM257 and LC756 were mixed with DCM and heated to 80 °C for 5 min and then cooled to 25 °C. Then, PETMP, EDDET, and I‐651 were added to the solution. DPA (diluted with DCM of 1: 50 v/v ratio) was added as a catalyst for the first‐stage Michael addition reaction to turn the system into a precursor with low crosslinking density. The reaction time was controlled for 30 min at room temperature. Then the precursor was used to fabricate the hollow fiber immediately.

### Precursor Synthesis and Fabrication of Hollow CLCE Mechanochromic Fibers—Fabrication of Hollow CLCE Mechanochromic Fibers

Hollow CLCE mechanochromic fibers were produced by depositing an extruded CLCE precursor solution on a mandrel, photocrosslinking after self‐assembly of CLCE. First, the precursor solution was transferred to the Y‐shaped needle syringe (5 mL, 10G). Then, the capillary glass tube (out diameter: 1 mm, length: 20 cm) was passed through the Y‐shaped needle at a constant speed, and the precursor solution was coated out of the capillary glass tube. Then, it was left for self‐assembling of liquid crystal units at 25 °C for 24 h. Once the cylindrically symmetric cholesteric order was stable after solvent evaporation, the precursor was crosslinked by 365 nm UV light with an intensity of 50 mW·cm^−2^ for 5 min. Subsequently, the capillary glass tube was etched off by hydrofluoric acid to obtain the hollow CLCE fibers. To balance the mechanical properties and mechanochromic sensitivity of hollow CLCE fibers, the molar ratio (f) of PETMP to EDDET was modified to 18%, 14%, 12%, 10%, 9%, and 8%, respectively. The formulation is shown in Table S1 (Supporting Information). The weight ratio (m) of chiral monomer LC756 to the total diacrylate monomer was set at 4.5, 6.0, and 7.5 wt.% for the hollow CLCEs fibers with red, green, and blue reflection color, respectively. The formulation is shown in Table S2 (Supporting Information).

### Characterization

POM images were taken with a Zeiss Primotech for microscopic observation. 2D‐WAXS experiment was conducted on a Xenocs Xeuss 2.0 Discover diffractometer in transmission mode. For the stretched fiber, a special fixture was used to get the strain of 38% before measurement. The reflection spectra were recorded using an ideaoptics PG‐2000 spectrometer. RGB values were analyzed by ImageJ software. The trend of the sample color was then plotted in CIE 1931 chromaticity diagram. Scanning electron microscopy (SEM) images were taken on HITACHI SU5000 scanning electron microscope analyzer. Differential scanning calorimetry (DSC) was performed using NETZSCH DSC 204 F1 with heat from ‐40 to 240 °C at a scanning rate of 10 °C min^−1^. Mechanical stress–strain experiments were performed by INSTRON 5967 instruments with CLCE fiber samples (Length 10 mm, diameter 1 mm) stretched at a strain rate of 50 mm min^−1^. Mechanical cycle experiments were performed by FANGYUAN YG001E instruments, the fiber was subjected to 100 cycles at the same rate ε_xx_ = 0‐0.38‐0, remaining for 180 s after each cycle, and σ_xx_ was continuously measured. The electrothermal color‐changing experiment was tested by ZHAOXINA DC POWER SUPPLY ZF‐3030 instrument (1 V 3.2A), Cr20Ni80 was used as a heating layer (diameter 0.7 mm), water was used as a heat transfer layer, hollow CLCE fiber was used as a color‐changing layer (length 0.7 mm). The average refractive index (n_avg_) experiment was performed by the Horoba UVISEL PLUS instrument (λ = 350–750 nm). The infrared thermal image using the FLUKE Ti400 IR FUSION TECHNOLOGY instrument with CLCE fiber samples (Length 10 mm, diameter 1 mm).

## Conflict of Interest

The authors declare no conflict of interest.

## Supporting information



Supporting Information

Supplemental Video 1

Supplemental Video 2

Supplemental Video 3

Supplemental Video 4

## Data Availability

The data that support the findings of this study are available from the corresponding author upon reasonable request.
