# Supplementary material for: Hollow Cholesteric Liquid Crystal Elastomer Fiber with Synergistically Enhanced Resilience and Mechanochromic Sensitivity
Source: Adv Sci (Weinh). 2025 Jun 25;12(34):e04487. doi: 10.1002/advs.202504487 (PMC12442651; doi:10.1002/advs.202504487)
Supplement: Supplementary file 1 — Supporting Information [file ADVS-12-e04487-s005.docx]

**Supporting Information**

**Hollow cholesteric liquid crystal elastomer fiber with synergistically enhanced resilience and mechanochromic sensitivity**

Wenwen Wang, Weijie Cheng, Feixia Liu, Kangyu Jia, Huihui Zhan, Chenguang Yang, Ke Liu, Qiongzhen Liu, Dong Wang^[[1]](#footnote-1)^

Key Laboratory of Textile Fiber and Products (Wuhan Textile University), Ministry of Education, Hubei International Scientific and Technological Cooperation Base of Intelligent Textile Materials & Application, Wuhan Textile University, Wuhan, 430200, China.

**Table S1** Chemical formulations and monomers molar ratio for CLCE hollow fibers.

| **Samples** | **Molar ratio of PETMP to EDDET** | **Mass of RM257 (mg)** | **Mass of LC756 (mg)** | **Mass of PETMP (mg)** | **Mass of EDDET (mg)** |
| --- | --- | --- | --- | --- | --- |
| 18%PETMP-CLCE | 18% | 268.8 | 12.1 | 17.92 | 38.08 |
| 14%PETMP-CLCE | 14% | 268.8 | 12.1 | 17.92 | 47.04 |
| 12%PETMP-CLCE | 12% | 268.8 | 12.1 | 17.92 | 54.88 |
| 10%PETMP-CLCE | 10% | 268.8 | 12.1 | 17.92 | 63.84 |
| 9%PETMP-CLCE | 9% | 268.8 | 12.1 | 17.92 | 72.8 |
| 8%PETMP-CLCE | 8% | 268.8 | 12.1 | 17.92 | 86.24 |

**Table S2** Chemical formulations and monomers molar ratio for CLCE hollow fibers with different original reflecting color.

| **Samples** | **Mass ratio of LC756 to RM257** | **Mass of RM257 (mg)** | **Mass of LC756 (mg)** | **Mass of PETMP (mg)** | **Mass of EDDET (mg)** | **Mass of I-651 (mg)** | **Mass of DPA (μl)** |
| --- | --- | --- | --- | --- | --- | --- | --- |
| 4.5%LC756-CLCE | 4.5% | 268.8 | 12.1 | 17.92 | 72.8 | 2.1 | 65 |
| 6.0%LC756-CLCE | 6.0% | 268.8 | 16.2 | 17.92 | 73.9 | 2.1 | 65 |
| 7.5%LC756-CLCE | 7.5% | 268.8 | 20.2 | 17.92 | 75.0 | 2.1 | 65 |

**Figure S1** Stress-strain curves of hollow CLCE mechanochromic fibers under different crosslinker ratio (f) from 8% to 18%.

**Figure S2** The gel content versus the crosslinker ratio.


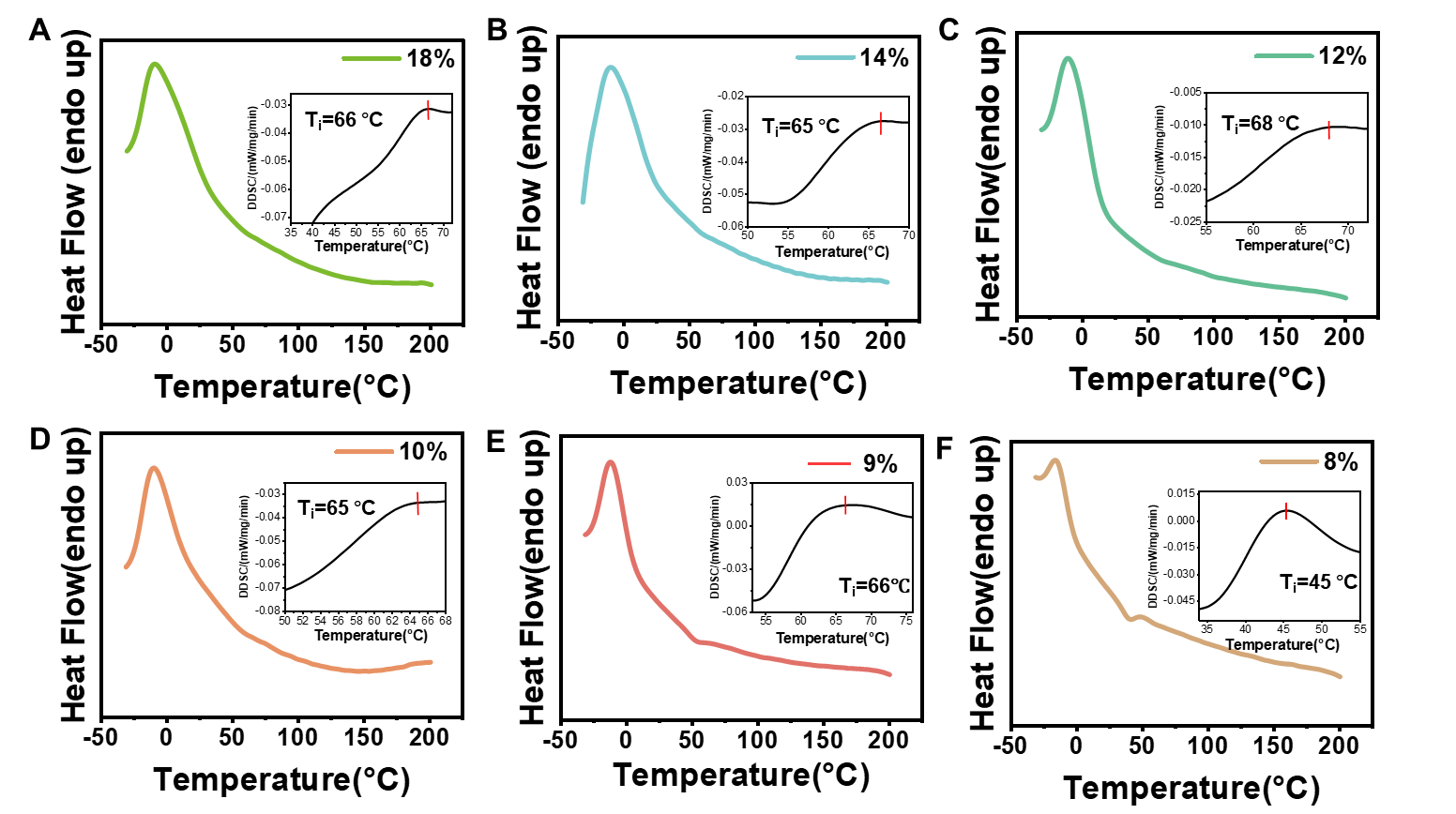


**Figure S3** DSC curves of these CLCE hollow fibers.


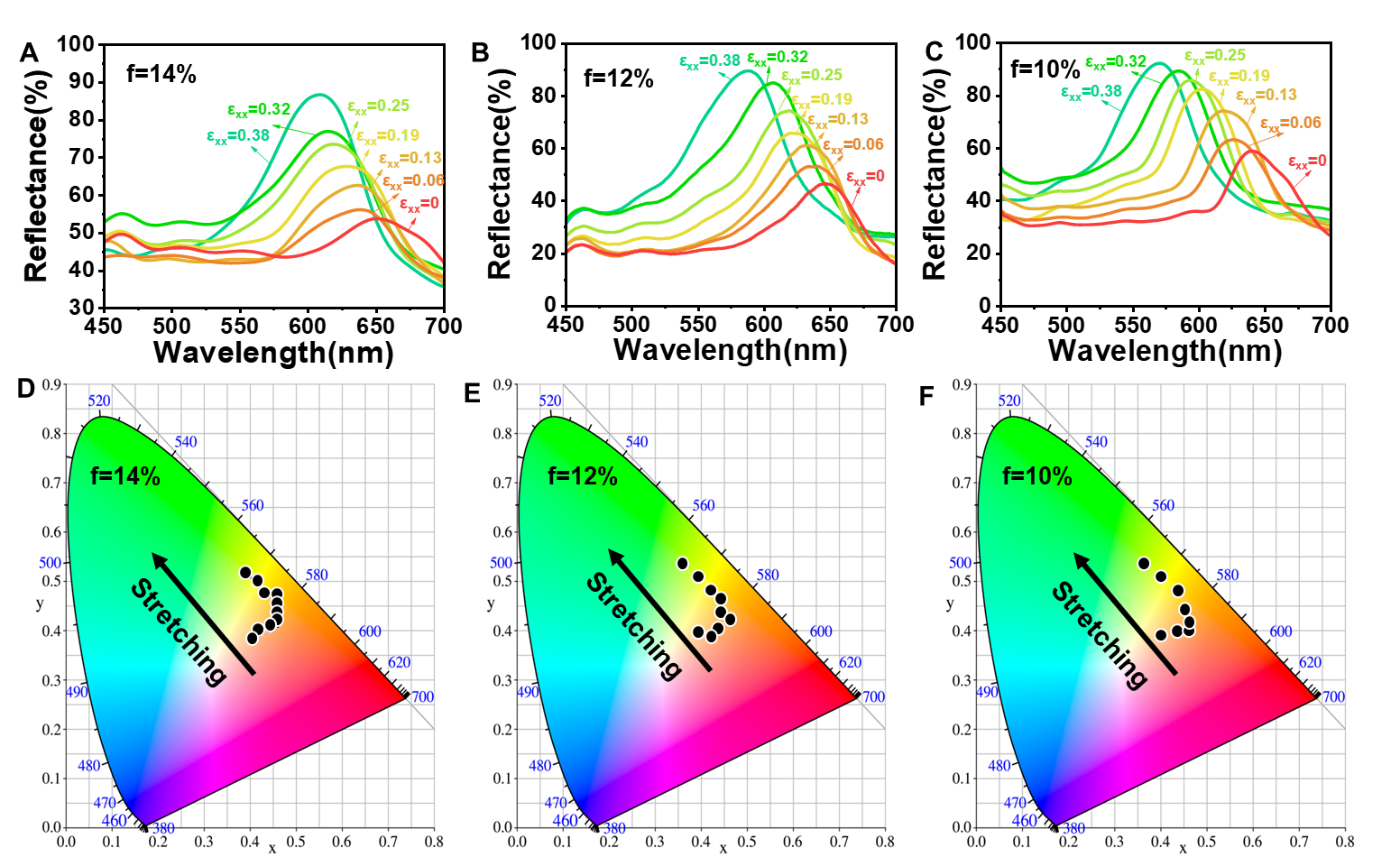


**Figure S4** (A, B, C) The reflection spectra of mechanochromic hollow fibers with different content of crosslinkers. (D, E, F) Change in the reflective color under a length strain of 0.00 to 0.38 on the CIE 1931 color space during stretching.

**
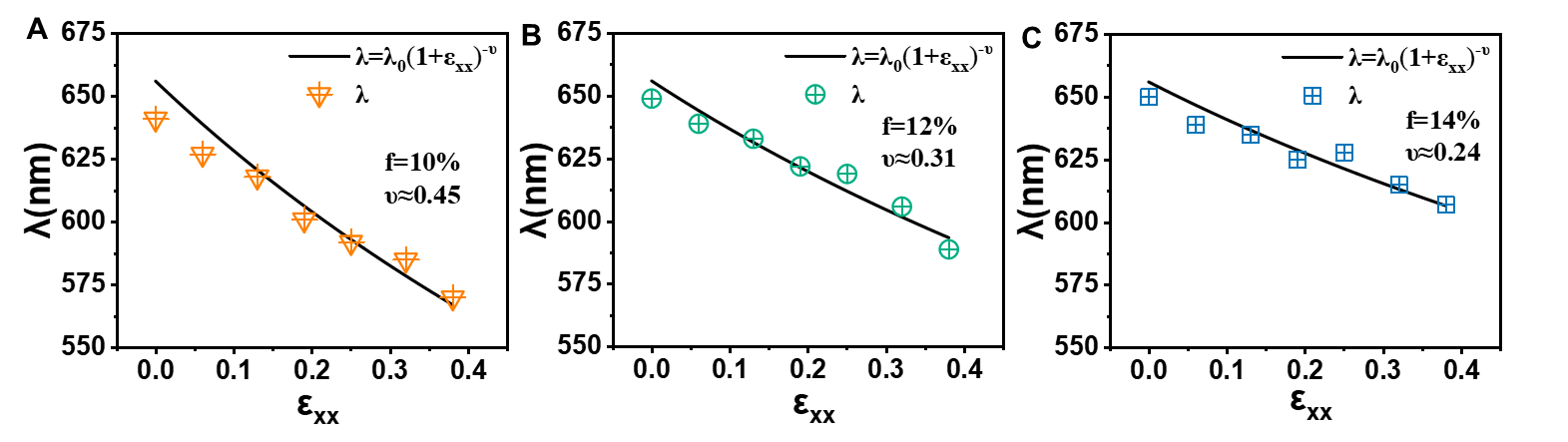
**

**Figure S5** Reflection wavelength 𝜆 versus ε_xx_ of different hollow CLCE fibers. The black line is the best fit of Equation (3) in the main paper.


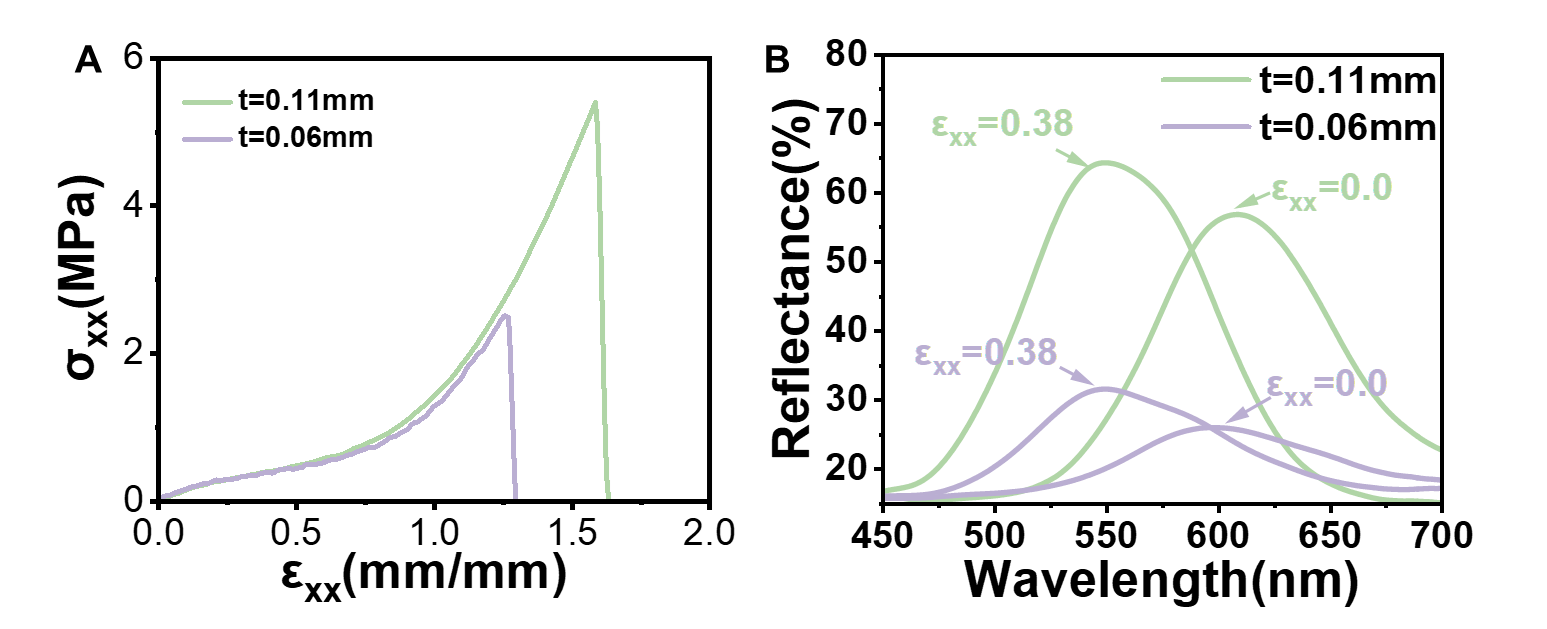


**Figure S6 (A)** The stress-strain curves and **(B)** reflection spectra of the hollow fibers with different wall thickness.

Numerical models have been established by Finite Element Method (FEM) for both solid and hollow fibers. The fibers both have a length of 10.00 mm. The solid fiber has a diameter of 1.00 mm, while the hollow fiber has an outer diameter of 1.00 mm, an inner diameter of 0.8 mm, and a wall thickness of 0.10 mm. The intrinsic Young's modulus of all fibers is 0.70 MPa, and the Poisson’s ratio is 0.495. One end of the fibers is fixed, and the other end is stretched along the X-axis by 5.00 mm.


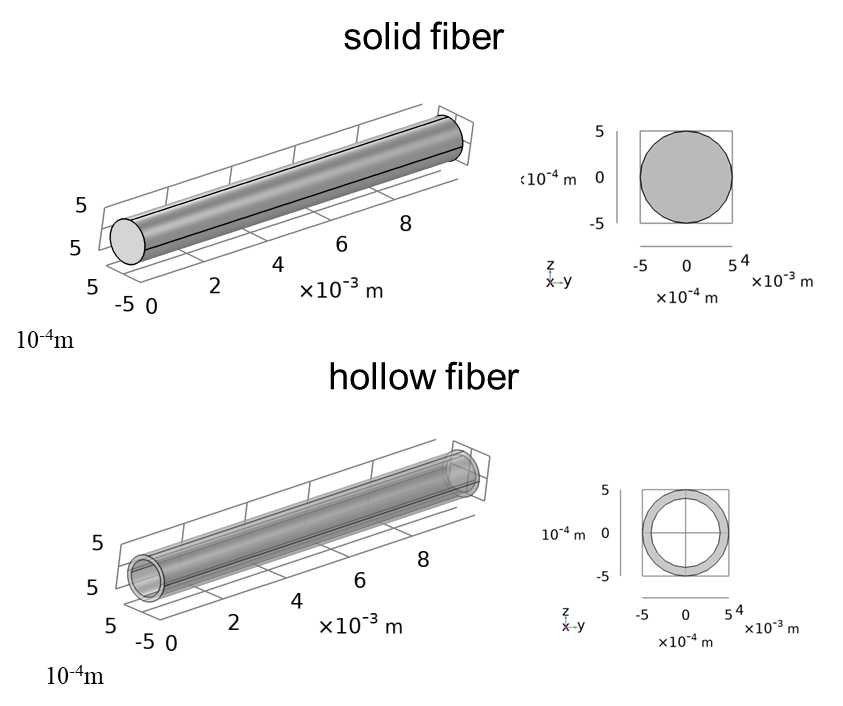


**Figure S7** The geometric models of solid and hollow fibers.


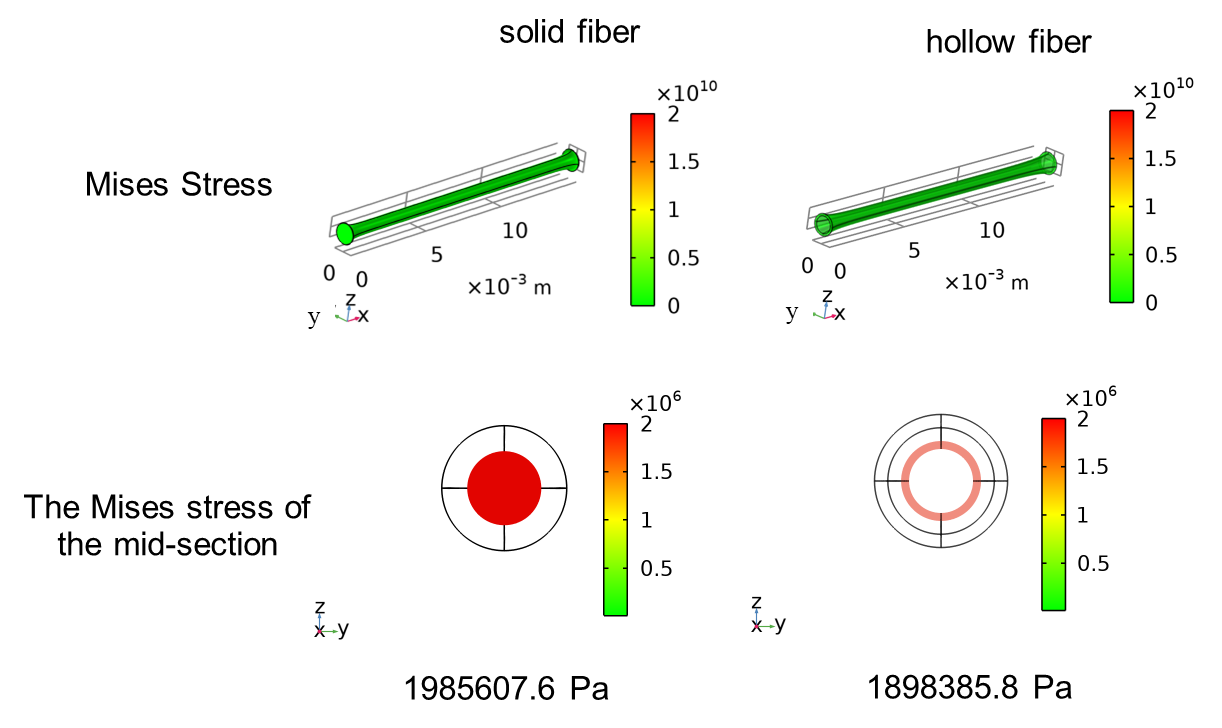


**Figure S8** The Mises stress of the whole fiber and mid-section fiber under external loading.

**Figure S9** The hysteresis loss of every stretching-recovery cycle.


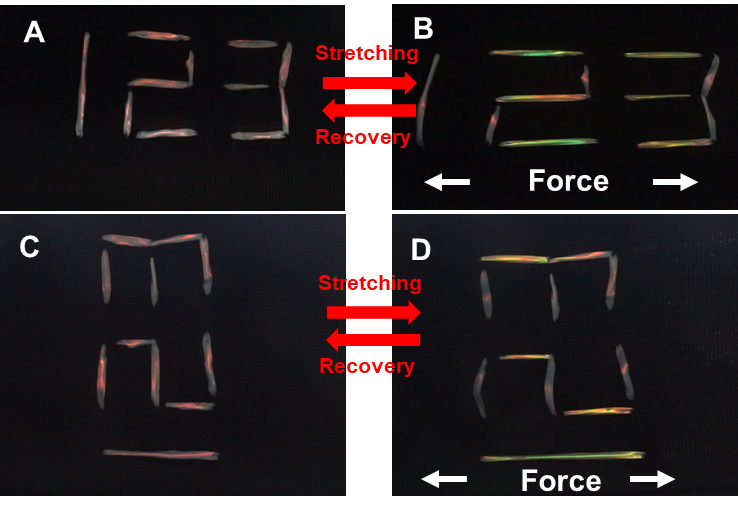


**Figure S10** The photographs of hollow fibers sewed up into the fabric with color change under stretching in different directions.

.
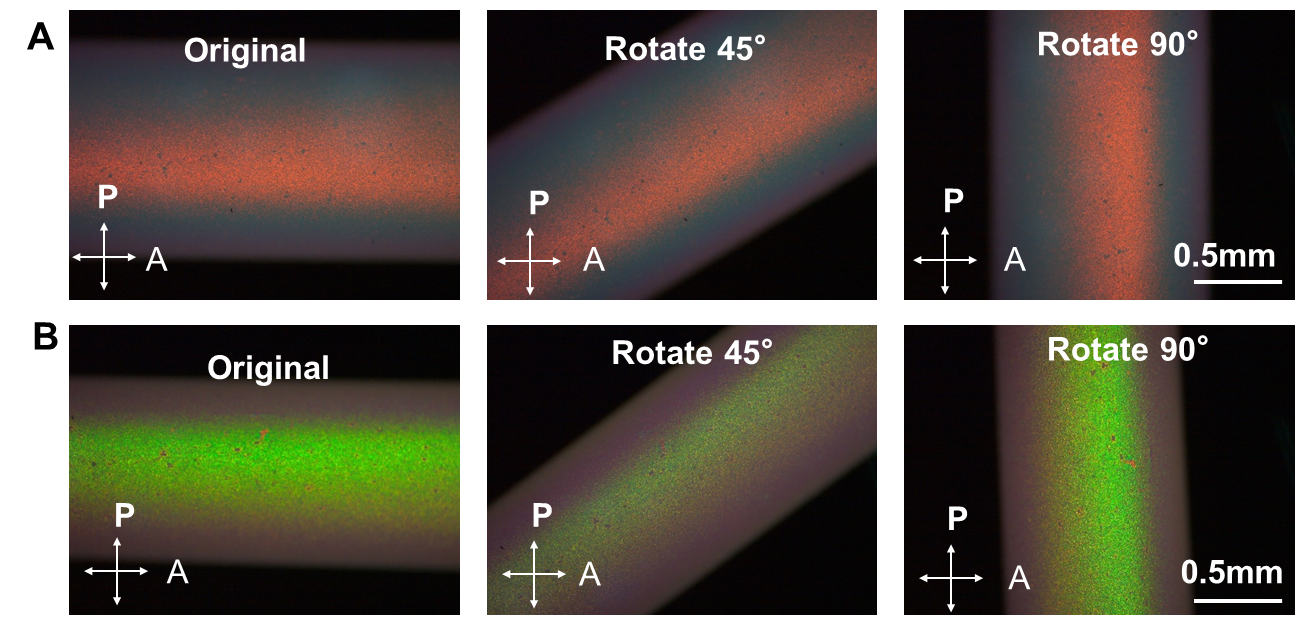

**f=0.7**

**f=0.8**

**f=0.9**

**ε_xx_=0.38**


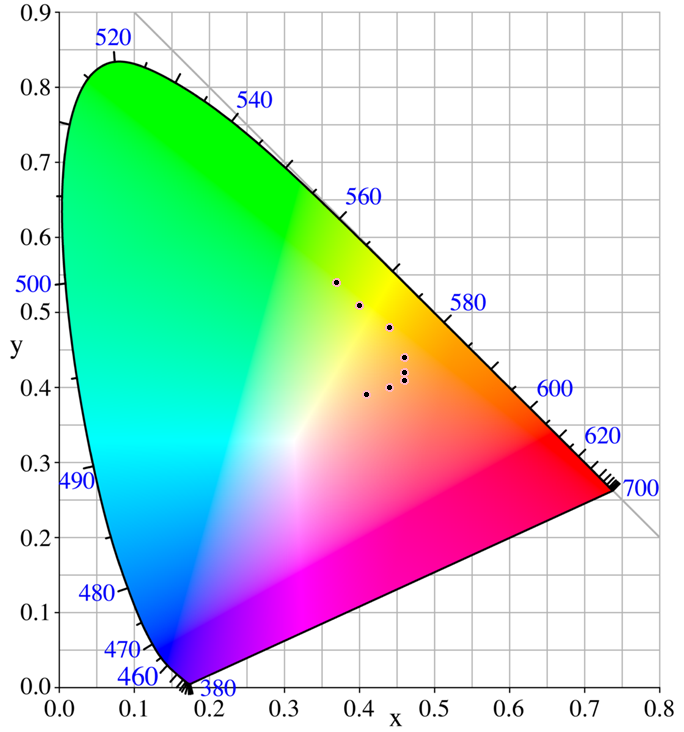


**f=0.9**


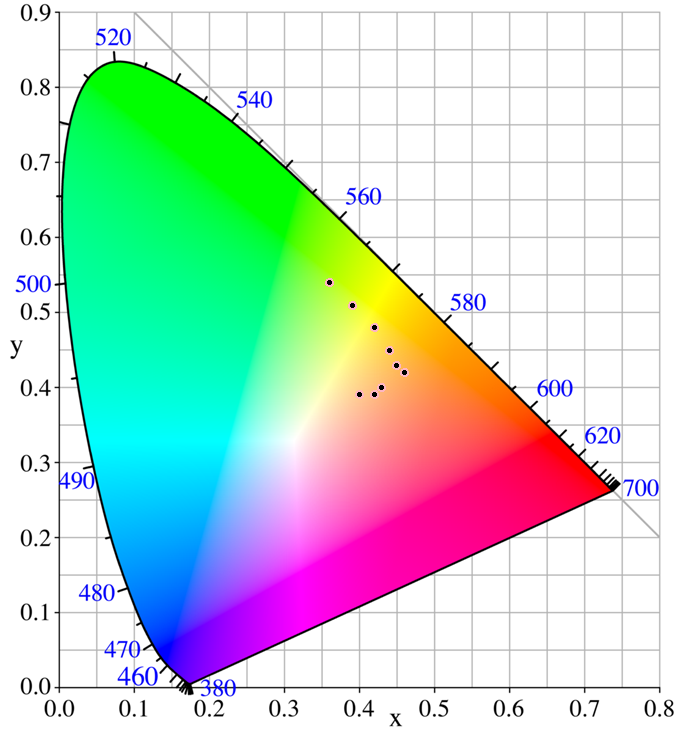


**f=0.8**


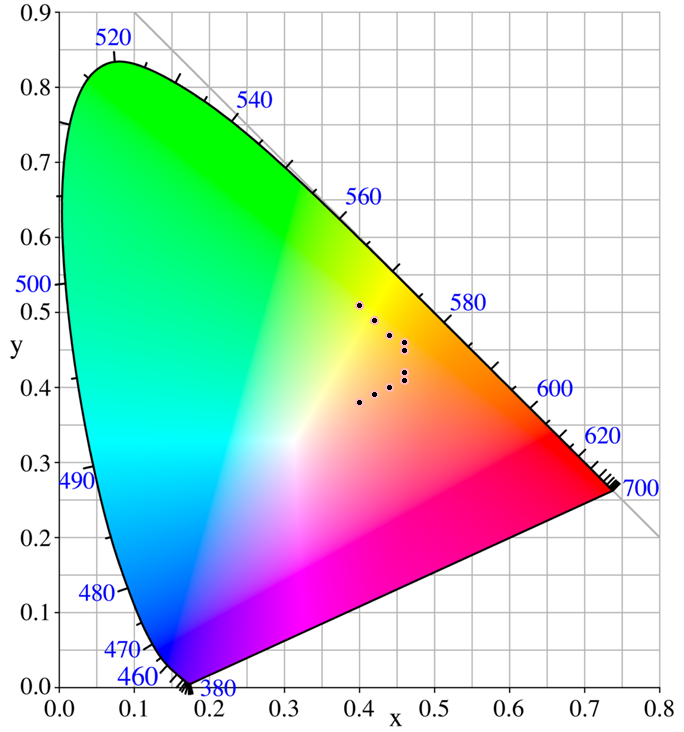


**f=0.7**

**Stretching**

**Stretching**

**Stretching**

**A**

**B**

**C**

**D**

**E**

**F**

**G**

**H**

**I**

**Figure S11** POM images of (A) the original red-reflecting CLCE hollow fiber and (B) the 38% strained CLCE hollow fiber at different angles.


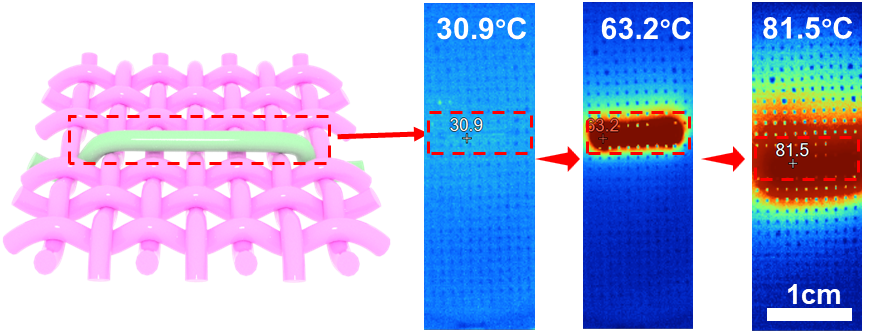


**Figure S12** Representation exhibiting the electrothermal effect of the composite fiber during voltage loading.


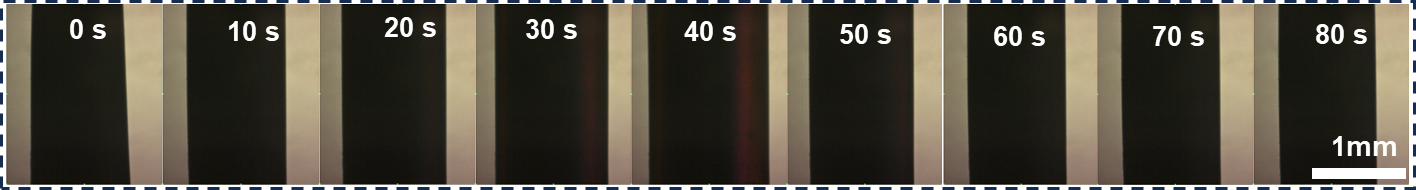


**Figure S13** The external diameter evolution of the composite fiber during power on and off.


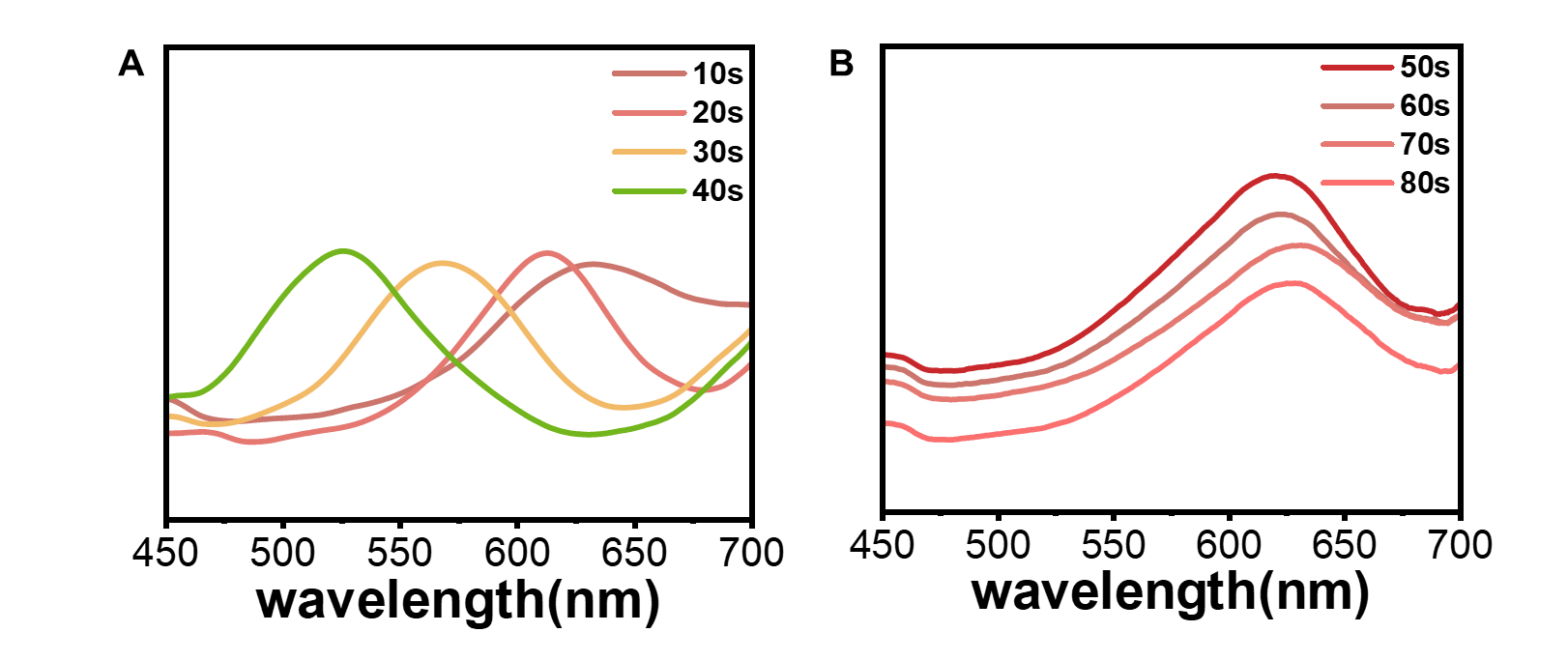


**Figure S14** The reflection spectra of the fiber surface during (A) power on and (B) power off.


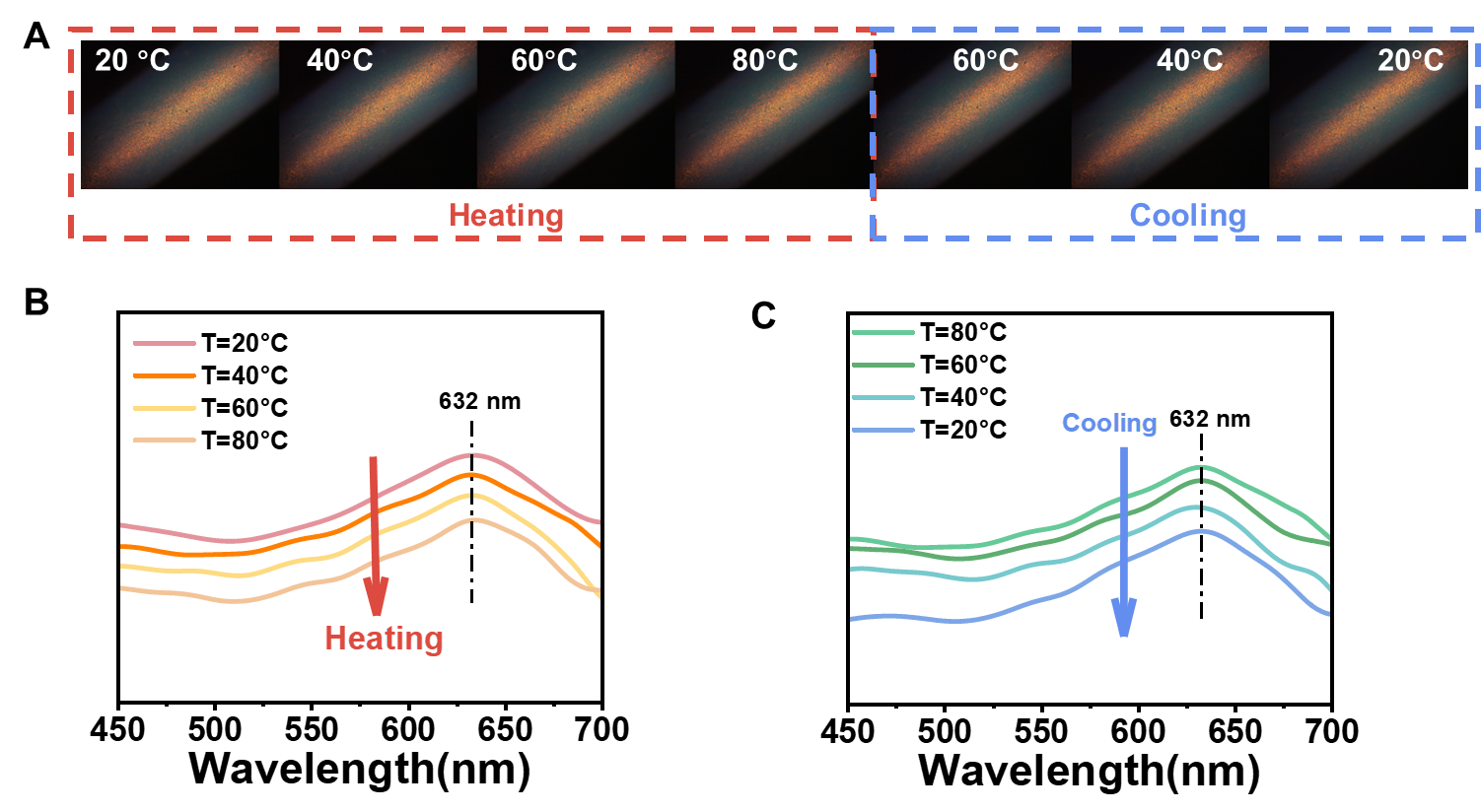


**Figure S15** (A) The photographs of the hollow CLCE mechanochromic fibers during heating and cooling. (B, C) The reflection spectra of the hollow CLCE mechanochromic fibers at different temperatures during heating and cooling.

1. Corresponding author. Email: wangdon08@126.com. [↑](#footnote-ref-1)
